# Operationalizing interdisciplinary research—a model of co-production in organizational cognitive neuroscience

**DOI:** 10.3389/fnhum.2013.00720

**Published:** 2014-01-16

**Authors:** Michael J. R. Butler

**Affiliations:** Work and Organisational Psychology Group, Aston Business School, Aston UniversityBirmingham, UK

**Keywords:** neurosciences, organizational cognitive neuroscience, organizational behavior, organizational sciences, interdisciplinary communication, co-production research

There is a biological turn in order to understand the underlying processes concerning markets and organizations. As part of the biological turn, in 2008, I wrote an article for the Journal of Consumer Behavior about neuromarketing and the perceptions of knowledge (Butler, 2008). The argument put forward in the article is that there are inter-related and potentially competing perspectives which combine to make up the biological turn. In order to conceptualize these varied perspectives I introduced a novel Neuromarketing Research Model. This commentary is concerned with updating the Model and using it to reveal some of the current intersections between society, organizations and the brain.

By taking this approach, I want to supplement David Waldman's opinion article in this special issue titled “Interdisciplinary research is the key.” Waldman (2013) argues that organizational sciences are rapidly coming together with neuroscience theory and methods to provide new insights into organizational phenomena, especially the larger problems facing organizations. I add to this argument by identifying specific points of how organizations and neuroscientists are coming together, and operationalize interdisciplinary research by proposing a new Model of Co-Production in Organizational Cognitive Neuroscience (OCN). OCN is defined as the application of neuroscientific methods to analyse and understand human behavior within the applied setting of organizations, which may be at the individual, group, organizational, inter-organizational, and societal levels. OCN draws together all the fields of business and management in order to integrate understanding about human behavior in organizations and to more fully understand social behavior (Butler and Senior, 2007).

I will re-introduce the purpose of the original Neuromarketing Research Model and state why it fits with this collection of papers, then I will briefly describe the Model in more detail. This will be followed by revising the Model to capture developments in OCN since 2008, and by using the updated Model to cohere different and fundamental themes and directions at the frontier of human neuroscience.

## Human modes of perception in organizational cognitive neurosciences

The purpose of connecting neuromarketing and the perception of knowledge was to address the perennial concern about the interconnection between research and practice, and the different perceptions about the development and application of knowledge about neuromarketing. This concern is implicit in the theme of society, organizations and the brain. Basic human neuroscience research in the field of management and organizations is likely to be applied to practitioners through knowledge exchange processes.

I used Jacob Bronowski and Kant to connect neuromarketing and the perception of knowledge. In 1967, Bronowski profoundly argued that it is pointless to talk about what the world is like when the modes of perception of the world which are accessible to us have changed so much (Bronowski, 1978). By the role of perception Bronowski (1978) took a Kantian view which argues that our knowledge of the outside world depends on our human modes of perception.

Nearly fifty years on from Bronowski's lecture series, our modes of perception have moved on again—neuroscience as a field of study has emerged. As a consequence, a Neuromarketing Research Model was proposed. The Model was developed from the work of Stokes (1997) and Tushman et al. (2007; Tushman and O'Reilly, 2007). Tushman et al. (2007; Tushman and O'Reilly, 2007) adapted Stokes' (1997) work to inform the debate about the role of business school research. Tushman et al. (2007; Tushman and O'Reilly, 2007) argue that unlike conventional academic disciplines which focus on basic disciplinary research (economics, psychology, and sociology) and consulting firms which focus on meeting clients' needs, business schools are about rigor and relevance. Whilst agreeing with Tushman et al.'s (2007; Tushman and O'Reilly, 2007) argument, their model is problematic because it compresses the range of business school activity into a narrow set of behaviors concerning research and its application.

In its place, the Neuromarketing Research Model interconnected different perceptions of neuromarketing knowledge. Basic research reporting satisfies the needs of academics and applied research reporting the needs of employers (Doherty, 1994). Media reporting is less definitive because it satisfies the needs of the target audience for the publication, which could be academic or practice-based. Similarly, power processes is less definitive because they satisfy the needs of dominant actors in the networks identified here by knowledge becoming ideological and biased in favor of particular actors through a conflictual process (Clegg and Palmer, 1996; Stiles, 2004). Waldman (2013) dedicates a section in his article to institutional and personal impediments hindering the application of neuroscience to his own area of expertise, leadership in organizations.

## A model of co-production in organizational cognitive neuroscience

**Figure 1:**
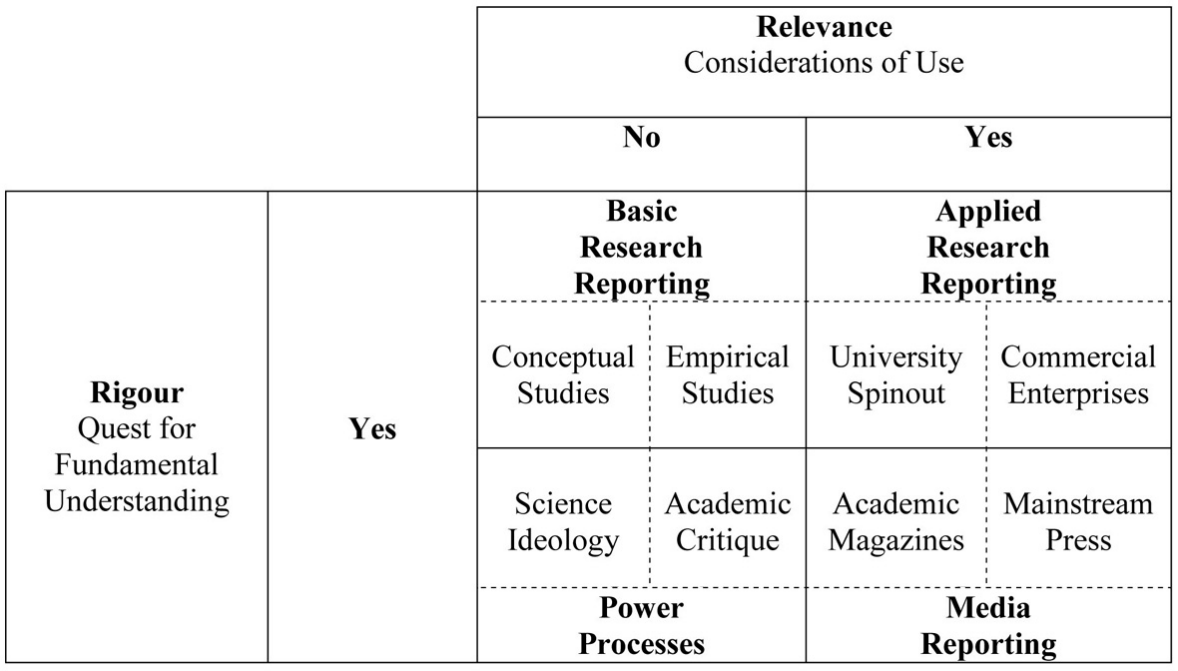
**Model of Co-Production in Organizational Cognitive Neuroscience**.

This commentary proposes a new Model of Co-Production in OCN because our modes of perception have moved on still further (Figure 1). The components of the original Model remain in place (in bold text). There are, however, four substantial changes. First, this commentary emphasizes the rigorous quest for understanding OCN rather than those which are less rigorous, in other words, the presentation of OCN work which reassures readers that appropriate methods and approaches have been adopted. Second, the new Model has additional elements to capture the emergent complexities at the intersection between society, organizations, and the brain. The new cells have dotted line divisions to indicate that they are sub-divisions of the four main quadrants described in the previous section: Basic Research Reporting, Applied Research Reporting, Media Reporting, and Power Processes. Third, because human neuroscience is being applied more widely across management and organizations, going beyond neuromarketing and neuroeconomics, the examples used in the following sections reflect this expansion of application. Fourth, the term “co-production” is used to describe the model.

Co-production is derived from a mode 2 approach to researching management and organizations (Gibbons et al., 1994). Knowledge is produced in the context of a real-world problem and the theoretical development is co-negotiated with practitioners. The Model of Co-Production in OCN reflects this intersection, highlighting both rigor and relevance, or the quest for fundamental understanding and the conditions of use. Indeed, university-organization relationships provide a productive setting for knowledge exchange research (Perkmann and Walsh, 2007). Waldman (2013) expresses this approach in his article stating he has had much more success at connecting with neuroscientists who combine the scientist-practitioner model, including establishing their own firms to produce applications to such maladies as attention deficit disorder and sleep apnea.

Waldman's (2013) point fits within the Applied Research Reporting quadrant of Figure 1, the university spinout cell. The quadrant as a whole emphasizes that practitioners are mindful of the need for scientific rigor and ethical considerations in human neuroscience work. Commercial success, whether a university spinout or another type of commercial enterprise, depends on clients having confidence in the results they are presented with and confidence comes from rigor and ethical practice (Brammer, 2004).

My focus is the intersection between Basic Research Reporting and Power Processes. In my original article, I noted that most attention is being given to basic research reporting because foundational research is currently being undertaken. The debates have become much more nuanced over the last 5 years and the new Model of Co-Production in OCN divides Basic Research Reporting into two further cells to take account of the wealth of conceptual articles and the growing empirical research. Conceptual debates are now appearing in established management and organization journals like the Journal of Management, and the journal web site has dedicated space to emphasize the emerging conversation about human neuroscience in the context of management (see Becker et al., 2011; Lee et al., 2012a).

In addition, special issues of highly regarded academic journals like the Leadership Quarterly capture specific themes at the intersection between organizations and the brain. Crucially, this allows conversations about OCN and leadership to involve both conceptual and empirical studies which include rigorous data collection and analysis (see Lee et al., 2012b). An illustrative empirical piece is Boyatzis et al. (2012), which examines the neural substrates activated in memories of experiences with resonant and dissonant leaders.

The intersection between the Basic Research Reporting and the Power Processes quadrants is crucial to the development of frontier research. As the number of published conceptual and empirical studies in the field of OCN grows, so does the academic critique of the OCN perspective. Rigorous and relevant debate advances OCN. This avoids knowledge, including emerging science theories like OCN, becoming ideological and biased in favor of particular actors through a conflictual process (Callon et al., 1986; Clegg and Palmer, 1996; Stiles, 2004).

Edwards (2013) introduces a realist critique of OCN. The argument being that it is important to consider how mental processes interact with “context” to produce social behavior. The Model of Co-Production in OCN is one manifestation of the interaction between the micro and macro levels. More generally, in the field of strategy implementation, my work explicitly acknowledges that different levels of change are co-evolving and dynamic (Butler, 2003; Butler and Allen, 2008).

Lindebaum and Zundel (2013a) rightly maintain that without explicit consideration of, and solutions to, the challenges of reductionism, the possibilities to advance leadership studies theoretically and empirically are limited. By reductionism they mean that neuroscientific approaches identify and analyze basic mechanisms that are assumed to give rise to higher order organizational phenomena, for instance, the way that inspirational leaders are identified and developed. In a lively exchange, Lindebaum (2012) and Cropanzano and Becker (2013) discuss the relative merits of neuro-feedback processes for the purpose of leader development and the ethical implications.

In terms of the Model of Co-Production in OCN, the previous discussion has an important implication for Basic Research Reporting—the danger of informing organizational practice inadequately and perhaps dangerously. As Edwards (2013) indicates, OCN can lend itself to over-interpretation, especially where scholars wish to find a simple and unique truth.

There is a similar implication for Media Reporting. The mainstream press can popularize ideas related to OCN and in doing so over-simplify complex research. Hannaford (2013), though, includes relevant research from leading institutions like the Max Planck Institute to support the argument in his newspaper article. Lindebaum and Zundel's (2013b) recent article in the academic magazine Times Higher Education also helps to re-balance popular perceptions of OCN.

## Concluding remarks

We are further along the lifecycle of the new field of study of OCN. Fugate (2007) argues that in order for OCN to become legitimized, it would be necessary to construct a behavioral model that would predict which stimuli provide the appropriate brain structure with the material it needs to accomplish its assigned task. We seem some distance from a behavioral model.

Nevertheless, this commentary has captured how research reporting within OCN is advancing, by proposing the Model of Co-Production in OCN. In particular, different themes and directions of research are found at the intersection between Basic Research Reporting and Power Processes. These debates, however, are also migrating into Applied Research Reporting and Media Reporting. This can only advance OCN. A variety of voices rigorously and relevantly debating OCN will advance this particular frontier in human neuroscience through the critique of emergent ideas.
